# BET inhibitor OTX015 targets BRD2 and BRD4 and decreases c-MYC in acute leukemia cells

**DOI:** 10.18632/oncotarget.4131

**Published:** 2015-05-14

**Authors:** Marie-Magdelaine Coudé, Thorsten Braun, Jeannig Berrou, Mélanie Dupont, Sibyl Bertrand, Aline Masse, Emmanuel Raffoux, Raphaël Itzykson, Marc Delord, Maria E. Riveiro, Patrice Herait, André Baruchel, Hervé Dombret, Claude Gardin

**Affiliations:** ^1^ Laboratoire de Transfert des Leucémies, Institut Universitaire d'Hématologie, University Paris VII, Paris, France; ^2^ Laboratory of Hematology, Hôpital Saint-Louis (Assistance Publique - Hôpitaux de Paris and University Paris VII), Paris, France; ^3^ Hematology Department, Hôpital Avicenne (Assistance Publique - Hôpitaux de Paris and University Paris XIII), Bobigny, France; ^4^ Leukemia Unit, Hematology Department, Hôpital Saint-Louis (Assistance Publique - Hôpitaux de Paris and University Paris VII), Paris, France; ^5^ Bioinformatics, Institut Universitaire d'Hématologie, University Paris VII, Paris, France; ^6^ Oncology Therapeutic Development, Clichy, France; ^7^ Oncoethix, Lausanne, Switzerland; ^8^ Department of Pediatric Hemato-Immunology, Hôpital Robert Debré (Assistance Publique - Hôpitaux de Paris and University Paris VII), Paris, France

**Keywords:** OTX015, BET inhibitors, c-MYC, HEXIM1, acute leukemias

## Abstract

The bromodomain (BRD) and extraterminal (BET) proteins including BRD2, BRD3 and BRD4 have been identified as key targets for leukemia maintenance. A novel oral inhibitor of BRD2/3/4, the thienotriazolodiazepine compound OTX015, suitable for human use, is available. Here we report its biological effects in AML and ALL cell lines and leukemic samples. Exposure to OTX015 lead to cell growth inhibition, cell cycle arrest and apoptosis at submicromolar concentrations in acute leukemia cell lines and patient-derived leukemic cells, as described with the canonical JQ1 BET inhibitor. Treatment with JQ1 and OTX15 induces similar gene expression profiles in sensitive cell lines, including a *c-MYC* decrease and an *HEXIM1* increase. OTX015 exposure also induced a strong decrease of BRD2, BRD4 and c-MYC and increase of HEXIM1 proteins, while BRD3 expression was unchanged. *c-MYC*, *BRD2, BRD3, BRD4* and *HEXIM1* mRNA levels did not correlate however with viability following exposure to OTX015. Sequential combinations of OTX015 with other epigenetic modifying drugs, panobinostat and azacitidine have a synergic effect on growth of the KASUMI cell line. Our results indicate that OTX015 and JQ1 have similar biological effects in leukemic cells, supporting OTX015 evaluation in a Phase Ib trial in relapsed/refractory leukemia patients.

## INTRODUCTION

Acute myeloid and lymphoid leukemias (AML and ALL) is a genetically complex and heterogeneous group of tumors associated with maturation arrest, expansion of abnormal hematopoietic progenitors, and abnormal remodeling of chromatin [1]. Recent reports describe a role for several members of the bromodomain and extraterminal (BET) protein family, including bromodomain-containing proteins BRD2, BRD3 and BRD4, in the maintenance of aberrant chromatin states in AML, ALL, myeloma and lymphoma [2], [3], [4], [5].

BRD2/3/4 activate transcription by binding to acetyl-modified lysine residues of histone tails [6]. As chromatin scaffolds, they recruit elements of the positive transcriptional elongation factor b (P-TEFb) complexes to RNA polymerase II (RNA Pol II) and initiate transcriptional elongation [7], [8]. Physiologically, P-TEFb-mediated elongation is regulated by the dynamic interplay of BRD4 and hexamethylene bisacetamide (HMBA) inducible protein 1 (HEXIM1) [9], [10]. BRD4 recruits P-TEFb to active promoters via its affinity to acetylated histones, while HEXIM1 inactivates P-TEFb via conformational changes and interaction of its C-terminal domain with 7SK snRNA [11]. In mice, c-MYC, a key regulator of cellular proliferation with oncogenic activity, has been shown to regulate P-TEFb-dependent pause release of Pol II [12].

BRD4 has been shown to drive oncogenetic processes by various mechanisms. The fusion protein BRD4-NUT is responsible for the aggressive NUT midline carcinoma [13], while interaction of BRD4 with acetylated NF-κB/RelA leads to constitutively active NF-κB, enhancing cancer cell proliferation [14]. BRD4 also directly interacts with NPM1, MDM2 and p53 proteins, notably via HEXIM1. Wild type NPM1 represses BRD4 by reducing its affinity to chromatin. This negative regulation is impaired in *NPM1*-mutated AML [15], [16]. Knockdown of *BRD4* in a shRNA screen demonstrated its critical role for maintenance of AML, as *BRD4* inhibition resulted in antileukemic activity *in vitro* and *in vivo* [2], [17]. BRD2 associates with transcriptional coactivators and corepressors, regulates expression of cyclin A and D1, and acts as an atypical kinase with intrinsic chaperone activity [18]. Overexpression of *BRD2* in murine B-cell progenitors induces a B-cell malignancy whose proteomic signature is reminiscent of human diffuse large B-cell lymphoma [19].

Inhibition of BET proteins thus constitutes an attractive therapeutic target. Pharmacologic BET inhibitors in development display significant activity in hematologic malignancies [20]. Treatment with the benzodiazepine-derived inhibitor JQ1 recapitulated anti-leukemic effects of shRNA-induced suppression of BRD4 in several AML cell lines, mouse models and primary patient samples [2], and has also been associated with potent cell growth inhibition, cell cycle arrest and cell senescence, and decrease of c-MYC in three murine multiple myeloma cell lines [4]. The small molecule BET protein inhibitors I-BET151 and I-BET762, belonging to the quinoline class of BET inhibitors, have also demonstrated *in vitro* activity in hematologic malignancies, including mixed lineage leukemia-related AML and multiple myeloma [21], [22]. BET inhibition by these agents results in preferential loss of BRD4 bound to super-enhancers and by consequence causes transcriptional repression of *c-MYC* [23].

OTX015, a thienotriazolodiazepine compound and a JQ1 analog, has been shown to inhibit binding of BRD2, BRD3, and BRD4 to acetylated histone 4 in a concentration-dependent manner, suggesting competitive inhibition, with IC50 values from 92-112nM (Kay Noel, American association for Cancer Research, AACR-NCI-EORTC International Conference on Molecular Targets and Cancer Therapeutics, Boston, MA, USA, oral communication, Oct 22, 2013). Here we studied the effects of OTX015 in a panel of leukemia cell lines, including the drug effects on cell growth, apoptosis and the expression of genes involved in the BRD2/3/4 signaling pathway. OTX015 was also evaluated *ex vivo* using primary cell samples from selected patients. OTX015 has entered clinical development in leukemia, with early results of an ongoing phase Ib study in advanced hematological tumors now available (Patrice Herait, AACR Annual Meeting, San Diego, LA, USA; Oral communication, Apr 04, 2014).

## RESULTS

### Effect of OTX015 on cell proliferation, cell cycle and apoptosis in leukemia cell lines

Cellular effects of OTX015 in various acute leukemia subtypes were evaluated. Cell viability after OTX015 exposure was assessed with the MTT assay in nine AML and four ALL cell lines. Significant growth inhibition, defined as a submicromolar IC50, was found in six of nine AML cell lines and all four ALL cell lines tested (Table 1). The K562 and KG1a AML cell lines were resistant to OTX015.

**Table 1:** IC50 in a panel of AML and ALL cell lines

AML cell line	Main genetic lesion	IC50 (nM)
K562	BCR-ABL	11342
KG1a	OP2-FGFR1	1342
HL60	NRAS Q61L	1306
HEL	JAK2 V617F	248
NB4	PML-RARa	233
NOMO-1	MLL-AF9	229
KG1	OP2-FGFR1	198
OCI-AML3	NPM1 A	60
Kasumi	AML1-ETO	17
**ALL cell line**	**Main genetic lesion**	**IC50 (nM)**
JURKAT	PTEN del	249
BV-173	BCR-ABL	161
TOM-1	BCR-ABL	133
RS4-11	MLL-AF4	34

Thirteen AML and ALL cell lines were exposed to OTX015 (0.01 nM to 10 μM). Cell proliferation was measured by the MTT assay at 72h and IC50 values were estimated. Experiments were performed in quadruplicates and means from three independent experiments are reported.

**Figure 1:**
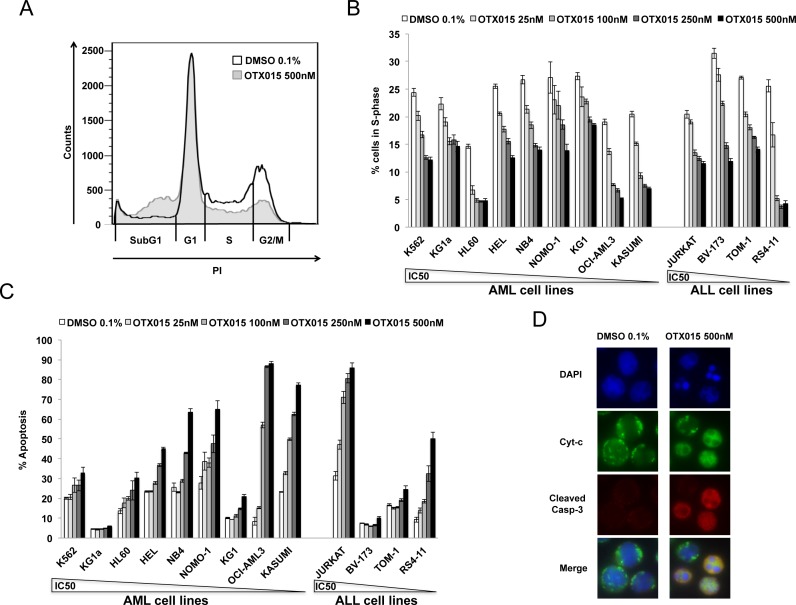
Effect of OTX015 on the cell cycle and apoptosis in AML and ALL cell lines Cell cycle alterations at 48h induced by increasing OTX015 doses (25nM-500nM) in leukemia cell lines: **A.** Representative flow cytometry overlay of the HEL cell line treated with 500nM OTX015 for 48h compared to 0.1% DMSO and **B.** percent cells in S-phase for all cell lines. Results are shown as mean +/− SEM from duplicates of three independent experiments. **C.** Apoptosis in AML and ALL cell lines after 72h exposure to increasing OTX015 doses (25nM-500nM). Apoptotic cells were defined as Annexin V+ with or without PI uptake. Results are shown as mean +/− SEM from duplicates of three independent experiments. **D.** Imunofluorescence for cytochrome *c* and activated caspase-3 in NOMO-1 cells after 72h exposure to 500nM OTX015 or 0.1% DMSO. Cytochrome *c* is shown in green, activated caspase-3 in red and nuclei are labelled blue. In non-apoptotic cells, cytochrome *c* (green) shows dotted staining localized to mitochondria, while no activated caspase-3 is detected, and in apoptotic cells, cytochrome *c* is released into the cytosol (green) and activated caspase-3 is localized in the cytoplasm (red). Merged images of apoptotic cells appear in yellow.

The effect of 500nM OTX015 exposure for 48h on the cell cycle resulted in decreased transition from G1 to S-phase in all 13 cell lines and a significant increase in cells in the sub-G1 phase in KG1a, KG1, HEL, KASUMI and JURKAT cell lines (Figure 1A, 1B and supplementary Figure 1).

Treatment with OTX015 at doses from 25 to 500nM for 72h induced significant apoptosis, as detected by Annexin V staining and PI uptake. At 500nM OTX015, 30-90% of cells were apoptotic in five of nine AML cell lines (HEL, NB4, NOMO-1, OCI-AML3, KASUMI) and 50-90% in two of four ALL cell lines (JURKAT and RS4-11; Figure 1C). Finally, 72h exposure to 500nM OTX015 induced mitochondrial apoptosis by cytochrome *c* release and caspase-3 activation (Figure 1D).

### Gene expression profiling of leukemic cell lines upon treatment with OTX015 and JQ1

**Figure 2:**
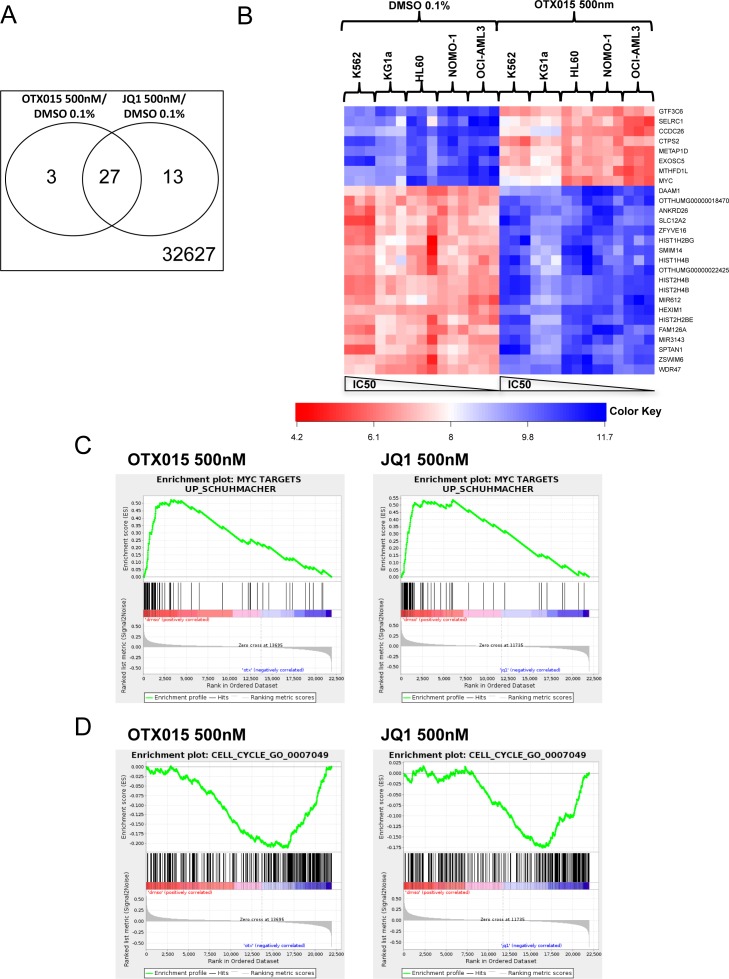
Molecular profiles after treatment with OTX015 and JQ1 in AML cell lines GeneChip Human Transcriptome Array HTA 2.0 (Affymetrix^®^) was performed for K562, KG1a, HL60, NOMO-1 and OCI-AML3 cells treated with either 500nM OTX015, 500nM JQ1 or 0.1% DMSO for 24h. Experiments were performed as triplicates. **A.** Venn diagram of signatures of 29 and 39 genes in the OTX015 vs. DMSO contrast and the JQ1 vs. DMSO contrast respectively. **B.** Heatmap of the JQ1 and OTX015 commune signature showing differently regulated genes in cell lines after treatment with OTX015 500nM compared to DMSO 0,1%. **C.** and **D.** *MYC* and cell cycle signatures enriched in all cell lines after treatment with OTX015 and JQ1.

The patterns of gene expression after treatment with 500nM OTX015 and 500nM JQ1 for 24h were similar for both drugs compared to controls (Figure 2A). Applying a cutoff of 1e-3 for the FDR and a cutoff of 0.9 for the log-fold-change in both comparisons, yielded signatures of 29 and 39 genes in the OTX015 *vs*. DMSO and the JQ1 *vs*. DMSO comparisons respectively. Among those genes, 27 were common to both signatures (Figure 2A, using a Fisher's exact test, the hypothesis of independence of both signatures was rejected; *p* = 2.2e-16). In cell lines (NOMO1, OCI-AML3, HL60, KG1a and K562), molecular signatures of OTX015 treatment were similar for the sensitive HL60, NOMO-1 and OCI-AML3 cell lines, including decrease or increase of *c-MYC* and *HEXIM1* expression (Figure 2B), whereas somewhat different expression patterns were noted for the less sensitive KG1a, and the resistant K562 cell lines.

Gene set enrichment analysis was performed for OTX015 or JQ1 *vs*. DMSO differential expression using the Gene Ontology Biological Process database (*n* = 794 genes). Similar enrichment in a representative *MYC* dependent gene set [24] and a representative cell cycle gene set (Biological process GO:0007079) [25] were observed in all cell lines for OTX015 and JQ1 respectively (Figure 2C and 2D).

### Effects of OTX015 on c-MYC, BRD2/3/4 and HEXIM1 in acute leukemia cell lines

**Figure 3:**
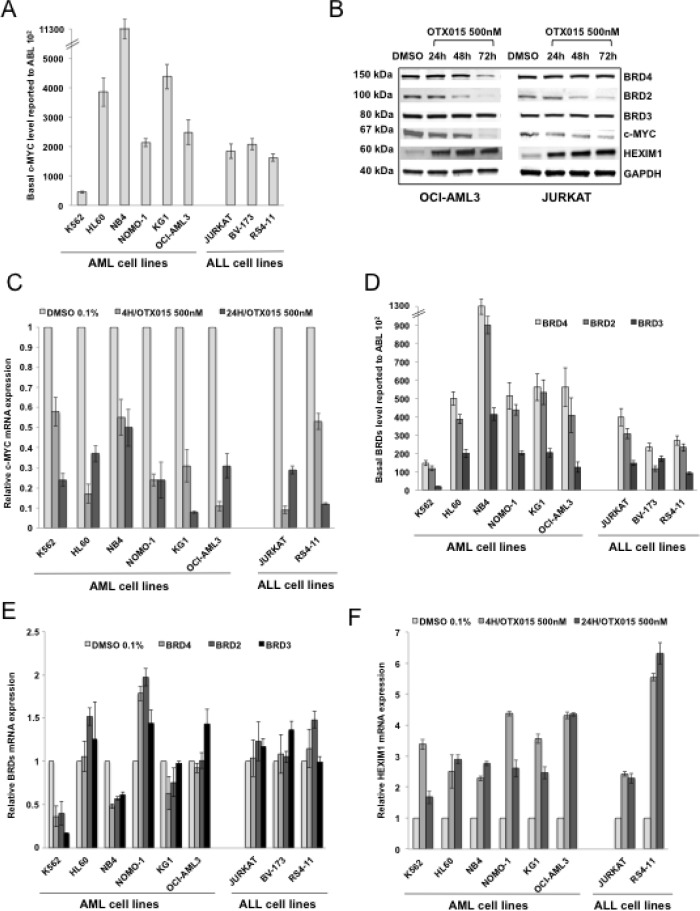
c-MYC, BRD2/3/4 and HEXIM1 expression in AML and ALL cell lines after OTX015 treatment **A.** *c-MYC* basal gene expression in AML and ALL cell lines determined by RT-qPCR, relative to ABL 10^2^. Results are shown as mean +/− SEM from duplicates of three independent experiments. **B.** Western blot showing c-MYC, BRD2/3/4, and HEXIM1 protein changes in OCI-AML3 and JURKAT cells treated with 500nM OTX015 for 24, 48 or 72h or 0.1% DMSO. GAPDH was used as a loading control. One representative experiment out of three is shown. **C.** RT-qPCR showing *c-MYC* decrease in AML and ALL cell lines after 4 and 24h exposure with 500nM OTX015, relative to *GAPDH* and normalized to 0.1% DMSO. Results are shown as mean +/− SEM from duplicates of three independent experiments. **D.** RT-qPCR showing *BRD4*, *BRD2*, and *BRD3* basal gene expression in leukemia cell lines, relative to ABL 10^2^. Results are shown as mean +/− SEM from duplicates of three independent experiments. **E.** RT-qPCR showing *BRD4*, *BRD2*, and *BRD3* mRNA expression levels after 48h exposure to 500nM OTX015 in leukemia cell lines, relative to *ABL* and normalized to 0.1% DMSO. Results are shown as mean +/− SEM from duplicates of three independent experiments. **F.** RT-qPCR showing *HEXIM1* mRNA increase in AML and ALL cell lines after 4h and 24h exposure with 500nM OTX015, relative to *GAPDH* and normalized to 0.1% DMSO. Results are shown as mean +/− SEM from duplicates of three independent experiments.

Based on the results of the expression arrays experiments, we first evaluated the effects on c-MYC protein and mRNA expression after exposure to OTX015 at 500nM for 4, 24, 48 or 72h of in a panel of AL cell lines. Basal *c-MYC* gene expression varied among cell lines, with lowest levels observed in *BCR-ABL*+ K562 cells and highest levels observed in the *PML-RAR*α rearranged NB4 cell line (Figure 3A). Following exposure to OTX015, c-MYC protein and mRNA expression was analyzed. We observed a c-MYC protein decrease to a variable extent as early as 24h after treatment in all cell lines tested, including AML cell lines (*NPM1*-mutated OCI-AML3, *BCR-ABL*+ K562, *PML-RAR*α*-*rearranged NB4, *MLL-AF9* fused NOMO1 and *NRAS*-driven HL60), and ALL cell lines (T-ALL JURKAT and *MLL-AF4* fused B-ALL RS4-11 cells) (Figure 3B, supplementary Figure 2A). In line with these results, *c-MYC* mRNA decreased ubiquitously after 4h and 24h OTX015 exposure in these cell lines as well as in the *OP2-FGFR1* rearranged KG1 AML cell line (Figure 3C). Treatment of these cell lines with 500nM JQ1 induced a decrease in c-MYC protein as seen with OTX015 at 24, 48 and 72h, as well as a similar *c-MYC* mRNA decrease at 48h in all cell lines tested (supplementary Figure 2B and 2C, respectively).

We also studied the effects of OTX015 exposure on gene and protein expression of BRDs. Among AML cell lines, basal gene expression levels of BRDs were lowest in the BCR-ABL*+* K562 cell line and highest in *PML-RAR*α rearranged NB4 (Figure 3D). After exposure to 500nM OTX015 for 48h *BRD2*, *BRD3* and *BRD4* mRNA expression strongly decreased in the K562 and NB4 cell lines but increased in HL60 and NOMO-1 cells (Figure 3E). Only mild variations of *BRD2*, *BRD3* and *BRD4* mRNA expression were observed in KG1, OCI-AML3, JURKAT, BV-173 and RS4-11. OTX015 induced a decrease in BRD2 protein in most cell lines, including OCI-AML3, JURKAT RS4-11, NB4, NOMO-1 and HL60 cells but not in K562 cells (Figure 3B and supplementary Figure 2A). In contrast, decreased BRD4 protein after OTX015 treatment was only seen in the OCI-AML3, NB4 and K562 cell lines. Finally, BRD3 protein levels were unmodified after OTX015 exposure in all cell lines analyzed (Figure 3B and supplementary Figure 2A). Compared to OTX015, treatment with JQ1 induced similar variations of BRD2, BRD3 and BRD4 proteins (supplementary Figure 2B).

The effect of OTX015 on *HEXIM1* expression was evaluated. *HEXIM1* mRNA expression increased both after 4 and 24h OTX015 exposure at 500nM in all cell lines tested (K562, HL-60, NB4, NOMO-1, KG1, OCI-AML3, JURKAT and RS4-11; Figure 3F). and was highest in OCI-AML3 and RS4-11 cell lines. Treatment with either OTX015 or JQ1 at 500nM (24-72h) yielded a similar increase in HEXIM1 protein levels after 24, 48 and 72h in OCI-AML3, JURKAT and RS4-11 cell lines but not in K562 cells (Figure 3B and supplementary Figure 2A and 2B).

Finally, basal mRNA expression levels of *c-MYC*, *BRD2*, *BRD3*, *BRD4* and *HEXIM1* did not significantly correlate with OTX015-induced loss of viability in any of the AML or ALL cell lines analyzed (supplementary Figure 3).

### *Ex vivo* effects of OTX015 in leukemic patient-derived samples

**Figure 4:**
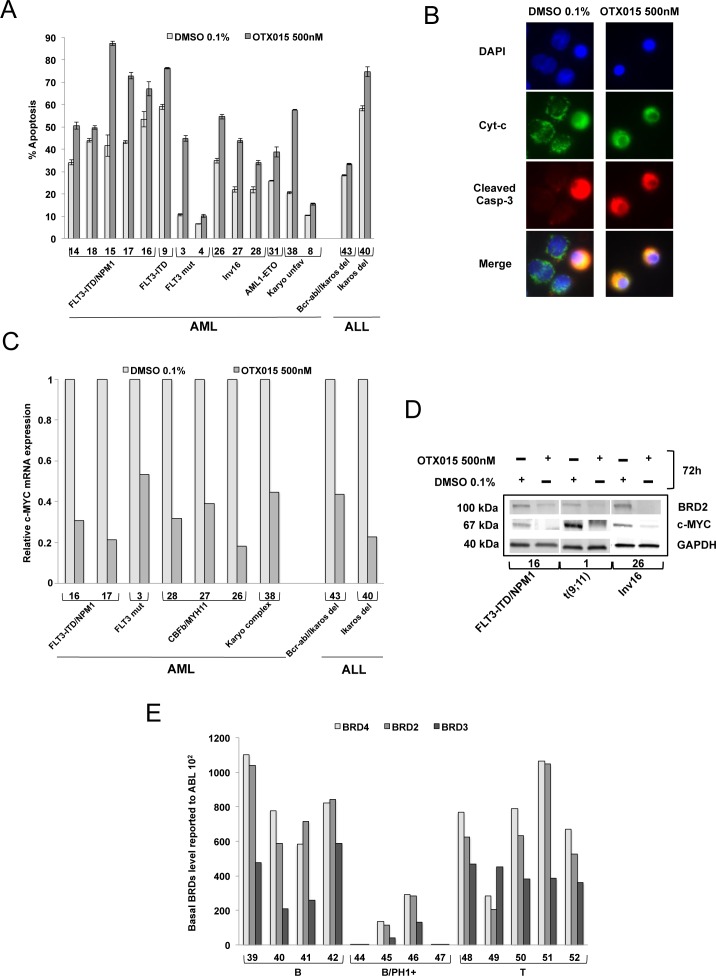
Induction of apoptosis, expression of c-MYC and BRD2 following OTX015, and basal expression of BRD2/3/4 in AML and ALL patient samples **A.** Bone marrow mononuclear cells were exposed to 500nM OTX015 for 72h. Apoptotic cells were defined as Annexin V+ with or without PI uptake. Results are shown as mean +/− SEM. **B.** BM cells from a patient with MLL-rearranged AML (*MLL-AF9*) (Patient 1, Table 2) showing cytochrome *c* (green), activated caspase-3 (red) and nuclei (blue). In non-apoptotic cells cytochrome *c* (green) shows dotted staining localized in the mitochondria while no activated caspase-3 could be detected, and in apoptotic cells cytochrome *c* is released into the cytosol (green) and activated caspase-3 is localized to the cytoplasm (red). Merged images of apoptotic cells appear in yellow. **C.** RT-qPCR showing *c-MYC* mRNA expression in nine AML and ALL patient samples after 72h exposure with 500nM OTX015 or 0.1% DMSO, relative to *ABL* normalized to 0.1% DMSO. **D.** Western blot showing BRD2, c-MYC and GAPDH expression in three AML patient samples exposed 72h to 500nM OTX015 or 0.1% DMSO *ex vivo*. **E.** RT-qPCR showing *BRD4*, *BRD2*, and *BRD3* basal gene expression levels in 13 ALL patient samples, relative to *ABL* 10^2^.

Apoptosis, mRNA and protein expression were evaluated in BM mononuclear cells obtained from representative newly diagnosed or relapsed ALL and AML patients for whom sufficient material for analysis was available (Table 2). Apoptosis induction by exposure to 500nM OTX015 for 72h was variable among the patient samples tested (Figure 4A). BM cells from 8 of 14 AML patients showed increased apoptosis ranging from 35-90% with OTX015 compared to control-treated cells (patients 3, 15, 17, 26, 27, 28, 31 and 38), while no or a mild increase in apoptosis was observed after OTX015 exposure in 6 of 14 patients (patients 4, 8, 9, 14, 16 and 18). BM cells from the two ALL patients tested showed no or a mild increase in apoptosis (patients 40 and 43).

**Table 2:** Patient characterization and analyses performed

Patient N°	Gender	Diagnosis	Karyotype	Molecular Biology	Apoptosis	mRNA	Protein
1	M	AML 2	46;XY; t(9;11)	MLLT3-MLL	no	no	yes
2	M	AML 2	46;XY	CEBP alpha	no	yes	no
3	F	AML 1	46;XX	FLT3 mut	yes	no	no
4	M	sAML	46;XY	FLT3 mut	yes	no	no
5	M	AML 2	46;XY	dup MLL	no	yes	no
6	F	AML 1	46;XX	dup MLL	no	yes	no
7	M	AML 5a	46;XY	FLT3 ITD/dup MLL	no	yes	no
8	M	sAML	46;XY; del(3)(q?),-7	FLT3 ITD / EVI1	yes	no	no
9	M	AML 5a	46;XY	FLT3 ITD	yes	no	no
10	F	AML 2	46;XX	FLT3 ITD	no	yes	no
11	M	AML	46;XY	FLT3 ITD	no	yes	no
12	F	AML	47;XX; der(10)(?)	FLT3 ITD	no	yes	no
13	F	AML 1	46;XX	NPM1 + FLT3 ITD	no	yes	no
14	F	sAML	46;XX	NPM1 + FLT3 ITD	yes	no	no
15	M	AML 4	46;XY	NPM1 + FLT3 ITD	yes	no	no
16	M	AML 5	46;XY	NPM1 + FLT3 ITD	yes	no	yes
17	M	AML 4	46;XY	NPM1 + FLT3 ITD	yes	no	no
18	M	AML 2	46;XY	NPM1 + FLT3 ITD	yes	no	no
19	F	AML 2	46;XX	NPM1	no	yes	no
20	F	AML 2	46;XX	NPM1	no	yes	no
21	M	AML 4	46;XY	NPM1	no	yes	no
22	M	AML 4	46;XY	NPM1	no	yes	no
23	F	AML 4	46;XX	NPM1	no	yes	no
24	M	AML 1	46;XY	NPM1	no	yes	no
25	M	AML 2	46;XY	NPM1	no	yes	no
26	F	AML 4eo	46;XX; inv(16)(p13q22)	CBFb/MYH11	yes	yes	yes
27	F	AML 4eo	46;XX; inv(16)(p13q22)	CBFb/MYH11	yes	yes	no
28	M	AML 4eo	46;XY; inv(16)(p13q22)	CBFb/MYH11	yes	yes	no
29	M	AML 4eo	46;XY; inv(16)(p13q22)	CBFb/MYH11	no	yes	no
30	F	AML 4eo	46;XX; inv(16)(p13q22)	CBFb/MYH11	no	yes	no
31	F	AML 2	46;XX; t(8;21)	AML1-ETO	yes	no	no
32	M	AML 2	46;XY; t(8;21)	AML1-ETO	no	yes	no
33	M	AML 2	46;XY; t(8;21)	AML1-ETO	no	yes	no
34	F	AML	Complex	ND	no	yes	no
35	F	AML	Complex	ND	no	yes	no
36	M	AML	Complex	ND	no	yes	no
37	M	AML	Complex	ND	no	yes	no
38	F	AML 5	Complex	ND	yes	yes	no
39	F	ALL-B	Complex	Ikaros del	no	yes	no
40	F	ALL-B	Complex	Ikaros del	yes	yes	no
41	M	ALL-B	46;XY	ND	no	yes	no
42	F	ALL-B	46;XX	ND	no	yes	no
43	M	ALL-B	46;XY; t(9;22)	BCR-ABL	yes	no	yes
44	M	ALL-B	46;XY; t(9;22)	BCR-ABL	no	yes	no
45	F	ALL-B	46;XX; t(9;22)	BCR-ABL	no	yes	no
46	M	ALL-B	46;XY; t(9;22)	BCR-ABL	no	yes	no
47	M	ALL-B	46;XY; t(9;22)	BCR-ABL	no	yes	no
48	M	ALL-T	46;XY; del(7)(p?)	HOX11L2	no	yes	no
49	F	ALL-T	Complex	ND	no	yes	no
50	F	ALL-T	Complex	ND	no	yes	no
51	M	ALL-T	Complex	ND	no	yes	no
52	M	ALL-T	ND	Calm/Af10	no	yes	no

We obtained CD34+ cells from six healthy donors. Apoptosis was assessed after 72h and OTX015 induced no significant apoptosis after 72h exposure in cells from 2 donors, while slight apoptosis was observed in 1 donor (supplementary Figure 4A). Clonogenicity assays showed no significant inhibition of colony growth for 2 patients while colony growth was inhibited in 1 patient (supplementary Figure 4B).

In line with our observations in cell lines, OTX015 also induced activation of caspase-3 and mitochondrial cytochrome *c* release in samples analyzed from three AML patients (Figure 4B).

After treatment with 500nM OTX015 for 48h, *c-MYC* mRNA decreased in the seven AML samples and two ALL samples (Figure 4C) evaluated. In three primary AML samples analyzed, c-MYC protein clearly decreased after 72h with 500nM OTX015, as did BRD2 protein (Figure 4D).

We studied basal BRD2/3/4 gene expression in 38 AML and 14 ALL patient samples of various subtypes. As observed in cell lines, gene expression levels were highly variable across AML and ALL subtypes with the lowest expression in bcr-abl rearranged ALL samples (Figure 4E, supplementary Figure 5 and Table 2).

### Simultaneous and sequential treatment of KASUMI cell line with OTX015, azacitidine and panobinostat

**Figure 5:**
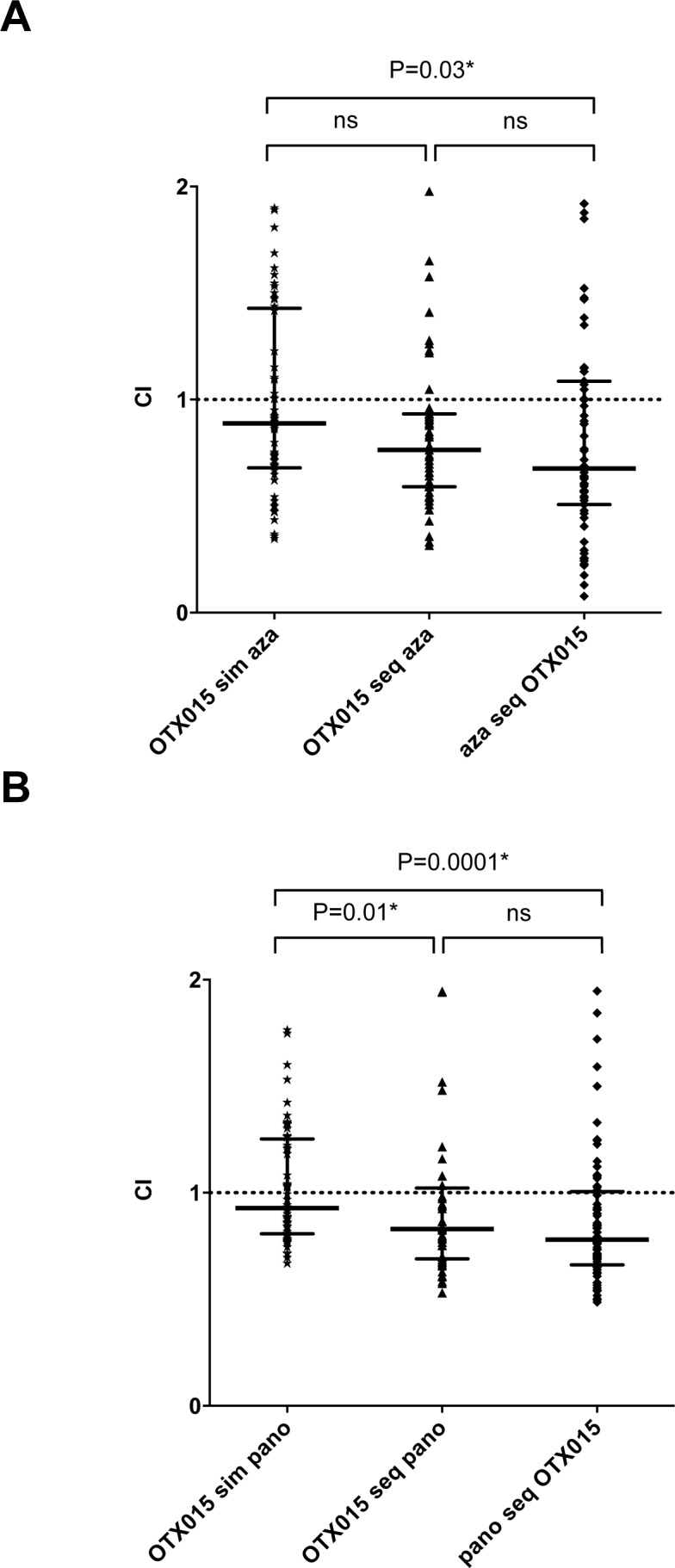
Simultaneous and sequential treatment of KASUMI cells with OTX015 and azacitidine and panobinostat show additional and synergic effects Combination Index (CI) versus Fractional Effect (FE) plots were calculated using CalcuSyn^®^ Software Version 2.1. Median CI of different dose combinations (simultaneous and sequential) were calculated for OTX 015 and azacitidine **A.** or OTX015 and panobinostat **B.**. CI values < 0.9 indicate synergy. Median CI values were calculated as median with interquartile range from three independent experiments and compared by the Mann-Whitney test.

We searched for synergistic effects of OTX015 on growth (7-600nM) if combined to other epigenetic modifying drugs: either azacitidine (123nM-10μM) or panobinostat (0.1-10nM). Synergy, was observed with both, simultaneous treatment of KASUMI cells by OTX015 and azacitidine (CI: 0.89) and sequential treatments, either OTX015 followed by azacitidine (CI: 0.76) or azacitidine followed by OTX015 (CI: 0.68) (Figure 5A). Synergy was significantly stronger when azacitidine was followed by OTX015 compared to simultaneous treatment with both drugs (*p* = 0.03). Significantly stronger synergy was observed during sequential treatments with either OTX015 followed by panobinostat or panobinostat followed by OTX015 (CI: 0.83 and 0.78 respectively) compared to simultaneous treatment (CI: 0.93; *p* = 0.0001 and 0.01 respectively) (Figure 5B).

## DISCUSSION

Treatment of acute leukemia remains a major clinical challenge, with disappointing long-term outcome of the majority of adult patients. AML is predominantly a disease of older patients, with overall survival of less than 10-20% in this age group [26]. Novel treatment strategies with acceptable toxicity are urgently needed to improve outcome. Here we demonstrate for the first time, that the oral BRD2/3/4 inhibitor OTX015, which is already in early clinical development in acute leukemia patients, has similar biological effects compared to JQ1, a prototypic BRD inhibitor, although restricted to *in vitro* use, in a broad range of acute leukemia cell lines and patient-derived leukemic samples. In most leukemic cell lines, relevant biological effects were observed in the submicromolar range with IC50 values and very similar for OTX015 and JQ1 [2]. In line, we observed cell cycle arrest in the G1/S transition and apoptosis rates as reported for JQ1.

The concentration range for which most biologic effects were observed can be achieved *in vivo*, as demonstrated in patients currently treated by OTX015 in the ongoing phase Ib clinical trial in refractory hematologic malignancies, in which promising antitumor activity has been seen in both leukemia and lymphoma patients (Patrice Herait, AACR Annual Meeting, San Diego, LA, USA; Oral communication, Apr 04, 2014). Available pharmacokinetics data indicate that this activity was detected with an OTX015 dosing and schedule giving plasma trough levels of approximately 250nM.

In line with reported outcomes for BET inhibitors JQ1 and I-BET in acute leukemia and other hematologic malignancies [2], [4], OTX015 also decreased c-MYC mRNA or protein as shown in the majority of the cell lines, as well as in primary acute leukemia samples. These observations confirm that c-MYC decrease is a major class effect of these drugs. A recent publication also confirmed that MYC is a key downstream target of BRD4-NUT [27], associated with an aggressive form of squamous cell carcinoma.

We show here that OTX015 exposure decreases BRD2 and BRD4 proteins, while BRD3 expression remained unaffected. Indeed it has been shown that that pharmacologic or shRNA inhibition of BRD4 protein expression leads to similar biologic effects [2]. More recently, inhibition of BRD2 by siRNA knockdown or treatment with JQ1 was shown to inhibit constitutive STAT5 activity in a large panel of acute leukemia and lymphoma cell lines [26]. In particular, strong synergy towards induced apoptosis was described when combined with tyrosine kinase inhibitors. It has also been demonstrated that the BET inhibitors I-BET151 and I-BET762 (currently under clinical investigation in NUT-midline carcinoma) exert their biologic activity via BRD2 decrease [20]. Our findings with OTX015 also suggest that BRD2 decrease participates in the anti-leukemic activity of OTX015. Further studies are needed to dissect the roles of BRD2 and BRD4 in OTX015 biologic effects [28].

Exposure to OTX015 or JQ1 increases HEXIM1 protein and mRNA expression [10]. Our findings of increased HEXIM1 gene expression after treatment with OTX015 are in line with those reported with either JQ1 or I-BET151, in multiple myeloma and ALL [3], [4], [22]. Overexpression of HEXIM1 leads to limited availability of active P-TEFb as a result of sustained BET inhibition in myeloma cells having a significant impact on the transcription of a large number of genes, including oncogenic MYC [29]. In line, OTX015 induced HEXIM1 overexpression may similarly intervene in the effects we observed in AL cell lines and AML patient samples.

OTX015 exposure induced growth inhibition, blocked cell cycle G1-S transition and caused apoptosis in acute leukemia cell lines and patient-derived leukemic samples, as previously reported for other preclinical BET inhibitors including JQ1 and I-BET151 [21], [30]. MLL fusion is associated with the BET family of acetyl-lysine recognizing chromatin adaptor proteins [21]. Similar to previous reports of BET inhibitors being highly active in MLL-fused leukemic cells, we show here that the MLL gene-rearranged NOMO-1 and RS4-11 leukemia cell lines are particularly sensitive to OTX015 [2], [3], [21], [31]. The *NPM1*-mutated cell line OCI-AML3 was also highly sensitive to OTX015, confirming results reported by Dawson et al [16] showing efficacy of I-BET151 in this cell line, as well as in a murine model of *NPM1*-mutated AML and in primary patient samples harboring *NPM1* mutations.

Interestingly, we were unable to identify any correlations between reductions of cell viability or induction of apoptosis and *c-MYC*, *BRD2/3/4* and *HEXIM1* expression levels in cell lines exposed to OTX015 (supplementary Figure 3). *c-MYC* decrease and *HEXIM1* increase induced by OTX015 was observed in all the cell lines analyzed, as reported with other BET inhibitors (supplementary Figure 6).

Recently, elegant studies in mouse models have shown that BET inhibition specifically targets recurrent genetic lesions in AML including *IDH2* and *FLT3-ITD* mutations, complete or interstitial chromosome 7 deletion, and inv(3)/t(3;3) associated with aberrant EVI1 expression [32], [33], [34]. Importantly, all of these genetic lesions are associated with a poor prognosis in patients treated with standard chemotherapy regimens [35]. BET inhibition has also been reported to target chemoresistant, ‘persister’ cells in T-ALL [36]. Thus, OTX015 might also be a promising therapeutic option for this subset of chemoresistant acute leukemia subtypes.

Epigenetic modifying drugs like the hypomethylating agent azacitidine and the histone deacetylase inhibitor panobinostat hold promise in the treatment of patients with AML uneligible for intensive treatment [37]. Furthermore it has recently been shown that targeting histone deacetylases in t(8;21) AML constitutes a potential molecular target [38]. In line, we have tested combinations of OTX015 with azacitidine and panobinostat in the t(8;21) KASUMI AML cell line. Simultaneous treatments mostly lead to additive effects while a strong synergy was observed with a sequential treatment with azacitidine followed by OTX015. This confirms earlier observations of synergic effects of combinations of the BET inhibitor JQ1 and AML chemotherapy (i.e. Cytarabine) [30].

On the other hand, to date, no mechanisms leading to resistance of BET inhibition have been identified. BCR-ABL driven K562 was the only cell line to be clearly resistant, but BCR-ABL T-ALL cell lines BV-173 and TOM-1 were highly sensitive to OTX015 treatment, which hints to other resistance mechanisms in K562 cells. Of note, the anti apoptotic gene *BCL2* was among the top ten of up-regulated genes in K562 cells as compared to OCI-AML 3 cells upon treatment with OTX015, indicating that up-regulation of anti apoptotic pathways could be implicated in resistance of K562 cells (supplementary Table 2). Nevertheless, somewhat different expression patterns for resistant K562 and less sensitive KG1a cells compared to other more sensitive cell lines were observed.

We have demonstrated that the BET inhibitor OTX015 decreases the expression of BRD2, BRD4 and c-MYC and increases the expression of the MYC negative regulator HEXIM1 as the BET-inhibitor JQ1 probe. Our data comfort the use of OTX015 in the ongoing phase 1b clinical trial in refractory hematologic malignancies including acute leukemias, and future studies in this indication.

## MATERIALS AND METHODS

### Cell lines and selection of primary patient cells

A panel of representative ALL and AML cell lines was purchased from Deutsche Sammlung von Mikroorganismen und Zellkulturen (DSMZ). ALL cell lines used were JURKAT (T-ALL), RS4-11 (MLL-AF4 B-precursor ALL), TOM-1, BV-173 (both Ph+ ALL). AML cell lines used were K562 (Ph+ CML in blast crisis), HL60 (*NRAS*-driven AML M2), NOMO-1 (*MLL*-*AF9*-driven AML), KG1 (*OP2-FGRF1* AML M6) and its more immature subtype KG1a, HEL (*JAK2* V617F), OCI-AML3 (*NPM1* and *DNMT3A* mutations), NB4 (*PML*-*RAR*α) and KASUMI (*AML1*-*ETO*). Cells were cultured in RPMI 1640 (Life Technologies) supplemented with 10% or 20% heat-inactivated fetal calf serum for ALL and AML lines respectively, 2mM L-glutamine, 100IU/mL penicillin, 100μg/mL streptomycin and HEPES, at 37°C with 5% CO_2_.

Mononuclear cells from the bone marrow (BM) of selected AML and ALL patients were isolated by Ficoll-Paque PLUS density gradient (Amersham Biosciences). Primary cells were maintained in IMDM (Life Technologies) supplemented with 10% heat-inactivated fetal calf serum, 2mM L-glutamine, 100IU/mL penicillin, and 100μg/mL streptomycin without growth factors, at 37°C with 5% CO_2_. Patients provided informed consent prior to BM aspiration at diagnosis, according to the Declaration of Helsinki. Approval for this study was obtained from the local institutional review board.

### Compounds

OTX015 (2-[(6S)-4-(4-Chlorophenyl)-2,3,9-trimethyl-6H-thieno[3,2-f][1,2,4]triazolo-[4,3-a][1,4]diazepin-6-yl]-N-(4-hydroxyphenyl) acetamide) was provided by Oncoethix (Lausanne, Switzerland) and (+)-JQ1 (tert-butyl 2-((6S)-4-(4-chlorophenyl)-2,3,9-trimethyl-6H-thieno[3,2-f][1,2,4]triazolo[4,3-a][1,4]diazepin-6-yl)acetate) was purchased from BPS Bioscience. Azacitidine (4-amino-1-β-D-ribofuranosyl-1,3,5-triazin-2(1*H*)-1) and panobinostat ([E]-N-hydroxy-3-4-((2-(2-methyl-1H-indol-3-yl)ethylamino)methyl)phenyl)-acrylamide were purchased from Selleckchem. All compounds were dissolved in dimethyl sulfoxide (DMSO; 1.0M stock solution) and stored at −80°C. Aliquots were thawed and used immediately for serial dilution in culture media. Control cells were incubated with 0.1% DMSO.

### MTT assay, apoptosis assessment and cell cycle analysis

For the MTT assay, cells were seeded in 24-well plates at 1×10^6^ per well and treated with a range of OTX015 concentrations 0.01nM-10μM for 72h. Cells were transferred to 96-well plates and incubated with 0.5mg/mL 3-(4,5-dimethylthiazol-2-yl)-2,5-diphenyltetrazolium bromide (MTT, Molecular Probes) in the dark at 37°C for 4h. Cells were then lysed with 25% sodium dodecyl sulfate (SDS) lysis buffer and absorbance was read at 570nm using a Promega Microplate Reader. Three independent experiments were run for each cell line and untreated cells were used as negative controls. The half maximal inhibitory concentration (IC50) values were calculated with Prism^®^ v6 software (GraphPad Inc).

For cell cycle analysis, 1×10^6^ cells were treated with a range of OTX015 concentrations 25-500nM for 48h then harvested, washed in PBS, and fixed in 70% ice cold ethanol. Cells were incubated with 100 μg/mL RNAse (Sigma) and stained with 25 μg/mL propidium iodide (PI; Becton Dickinson) for 30 minutes at 37°C.

For apoptosis analysis, 1×10^6^ cells derived from patients or cell lines were resuspended in 1 ml culture medium and treated with OTX015 for 72h. Apoptotic cells were detected using a FACSCalibur flow cytometer (Becton Dickinson). Cells were stained with 5μg/mL PI and Annexin-V-FITC (Becton Dickinson) according to the manufacturer's instructions for 15 minutes at room temperature. Apoptotic cells were defined as Annexin V+ with or without PI uptake.

Cell cycle distribution and apoptosis were determined by cytofluorometric analysis using a FACSCalibur flow cytometer (Becton Dickinson) and analyzed with the FlowJo flow cytometry software (TreeStar Inc).

### Immunofluorescence

Cells were allowed to adhere to polylysine-L slides (Thermo Scientific) and analyzed using an Image-iT^®^ Fixation/Permeabilization Kit (Life Technologies). Briefly, cells were fixed for 15 minutes with 4% paraformaldehyde in PBS (pH7.3) at room temperature, then permeabilized with Triton X-100 0.5% for 15 minutes and blocked for 1 hour with 3% BSA in DPBS (pH7.4). Cells were incubated with cleaved caspase-3 antibody (rabbit monoclonal Ab 9664; Cell Signaling Technology) or cytochrome *c* antibody (mouse monoclonal Ab 556432; Becton Dickinson) overnight at 4°C. Secondary antibodies were goat anti-rabbit and goat anti-mouse IgG coupled with Alexa 568 (red) or Alexa 488 (green) fluorochromes (Life Technologies) incubated at 37°C for 2h. Nuclei were counterstained with DAPI (Vector). Images were acquired by immunofluorescence microscopy on a Zeiss Axiovert microscope with a Plan-Apochromat X63 N.A.1.4 oil immersion objective using the Axiovison software v4.2 (Carl Zeiss).

### Quantitative-real time polymerase chain reaction (RT-qPCR)

Total RNA after extraction with TRIzol (Invitrogen) was titrated to 1μg/μL with a NanoDrop 2000c UV-Vis spectrophotometer (Thermo Scientific) and stored at −80°C. Complementary DNA (cDNA) was synthesized from 1μg RNA with a reverse transcriptase M-MLV Kit^®^ (Life Technologies) using Random Hexamer Primers (Thermo Scientific). RT-qPCR reactions (*BRD2, BRD3, BRD4, c-MYC, HEXIM1, GAPDH* and *ABL*) were performed in 25 μl from one- tenth of the cDNA volume (100 ng RNA), using a thermocycler ABI7900HT (Life Technologies) with TaqMan reagent or a StepOnePlus (Life Technologies) with SYBR Green reagent (Roche) in standard mode (1 cycle of 2 minutes at 50°C then 10 minutes at 95°C followed by 50 cycles of 15 seconds at 95°C then 1 minute at 60°C) with a supplementary melting curve step for SYBR Green assays. Primers (Eurogentec) are listed in supplementary Table 1. mRNA levels were normalized to *ABL* control gene.

### Gene expression profiling

NOMO-1, OCI-AML3, HL60, KG1a and K562 cells were treated for 24 hours either with 500nM OTX015, 500nM JQ1 or 0.1% DMSO (vehicle). Total RNA was extracted from corresponding cell lines. 500ng of RNA of each sample was processed using the WT PLUS Amplification and Labeling Kit according to the manufacturer's instructions, and analyzed with the GeneChip Human Transcriptome Array HTA 2.0 Array (Affymetrix^®^). This covers 44,699 coding transcripts, and 22829 non-coding transcripts. The WT plus Reagent Kit generates amplified and biotinylated sense-strand DNA targets from total RNA. Each sample was hybridized on the array, washed, and stained with the Affymetrix^®^ Fluidics Station 450. They were scanned with the Affymetrix^®^ GeneChip Scanner 3000 7G using the Command Console software (Affymetrix^®^) then analysed using the Affymetrix^®^ rma-sketch routine. A total of 72 chips were included in the quality control and normalization. OTX015-treated (500nM) (*n* = 3 replicates, time point 24h), JQ1-treated cells (500nM) (*n* = 3 replicates, time point 24h), DMSO 0.1%-treated cells (*n* = 3 replicates, time point 24h) and untreated control cells (*n* = 3 replicates, time point 24h).

Differential gene expression was assessed using the Bioconductor limma library on annotated coding transcripts (*n* = 32670). The multiple hypothesis testing issue was adressed using the False Discovery Rate (FDR) approach of Benjamini and Hochberg [39]. Metabolic pathways analysis was performed using the GSEA v2.0 software with 1000 gene set permutations.

### Immunoblotting

Protein was extracted from 7×10^6^ cells exposed to either OTX015, JQ1 or 0.1% DMSO; 30μg was loaded on SDS-polyacrylamide gels using 4-15% gradient gels (Bio-Rad) and transferred to nitrocellulose membranes using a Mini Trans-Blot Electrophoretic Transfer cell (Bio-Rad). Membranes were blocked with blocking buffer (LICOR) and incubated with the primary antibody overnight at 4°C: anti-BRD4 (#5716-1, Epitomics), anti-BRD3 (ab56342, AbCam), anti-BRD2 (ab37633, AbCam), anti-c-MYC (#sc-764 [N262], supplier), anti-HEXIM1 (#sc-365413, Santa Cruz) or anti-GAPDH (#398600, Invitrogen). Secondary antibodies were goat anti-rabbit InfraRedDye 680RD or goat anti-mouse InfraRedDye 800CW (LICOR), incubated for 2h at room temperature. Bands were visualized using a LiCor Odyssey scanner. For BRD2, membranes were secondarily stained with goat (BRD2) anti-rabbit peroxidase-labeled or goat anti-mouse (GAPDH) peroxidase-labeled secondary antibody (Bio-Rad) and visualized with an enhanced chemiluminescence detection system (ECL or ECL plus, GE Healthcare).

### Drug-dose-response experiments and CalcuSyn^®^ analysis

In combination experiments of OTX015 with azacitidine or panobinostat, compounds were added simultaneously or sequentially and relative cell numbers were determined at 48h with MTT assays. IC50 values were calculated with Prism^®^ v6 software (GraphPad Inc) at various concentrations of OTX015, azacitidine and panobinostat. Combination index (CI) values were calculated with *CalcuSyn*^®^ Version 2.1 software (Biosoft, Cambridge, UK) according to the Chow and Talalay model [40].

### Statistical analysis

Correlations between gene expression and cell viability were assessed using a Pearson's correlation coefficient. Statistically significant differences in CI for different drug combinations were calculated from medians compared by the Mann-Whitney U test. A heatmap for genes of interest was computed with the R software (www.*cran.r-project.org*).

## SUPPLEMENTARY MATERIALS TABLES AND FIGURES


