# Supplementary material for: BET inhibitor OTX015 targets BRD2 and BRD4 and decreases c-MYC in acute leukemia cells
Source: Oncotarget. 2015 May 14;6(19):17698–712. doi: 10.18632/oncotarget.4131 (PMC4627339; doi:10.18632/oncotarget.4131)
Supplement: Supplementary file 1 [file oncotarget-06-17698-s001.pdf]

BET inhibitor OTX015 targets BRD2 and BRD4 and decreases c-MYC in acute leukemia cells

Supplementary Material

Supp Table 1*: RT-qPCR primers and probes.*

**TAQMAN®**		
**c-MYC**	Forward primer	5'-GGATTTTTTTCGGGTAGTGGAA-3'
	Reverse primer	5'-TTCCTGTTGGTGAAGCTAACGTT-3'
	Probe	5'-FAM-CTCCCGCGACGATGCCCCT-TAMRA-3'
**BRD4**	Forward primer	5'-CCCTGAAGCCGTCCACACT-3'
	Reverse primer	5'-TTCTCAGCTTGAGGTTTCCTTTTC-3'
	Probe	5'-FAM CGCTATGTCACCTCCTGTTTGCGGA TAMRA-3'
**BRD3**	Forward primer	5'-ACATGCAGAATGTGGTGGTGAA-3'
	Reverse primer	5'-CGTCCACGGGCTGGTAGA-3'
	Probe	5'-FAM ACGCTCTGGAAACACCAGTTCGCCT TAMRA-3'
**BRD2**	Forward primer	5'-CCCGACGAGATTGAAATCGA-3'
	Reverse primer	5'-CCGCAAACAGGAGGTGACATA-3'
	Probe	5'-FAM TTGAGACCCTGAAGCCGTCCACACTG TAMRA-3'
**ABL**	Forward primer	5'-TGGAGATAACACTCTAAGCATAACTAAAGGT-3'
	Reverse primer	5'-GATGTAGTTGCTTGGGACCCA-3'
	Probe	5'-FAM CCATTTTTGGTTTGGGCTTCACACCATTTAMRA-3'
**SYBER Green®**		
**HEXIM1**	Forward primer	5'-AAGGACTAGCTAAAGGCGTCAC-3'
	Reverse primer	5'-TGGCTAGTAGAGTCCTCGAAGTTT-3'
**c-MYC**	Forward primer	5'-CGACTCTGAGGAGGAACAAGAA-3'
	Reverse primer	5'-GGATAGTCCTTCCGAGTGGA-3'
**GAPDH**	Forward primer	5'-GATCCCTCCAAAATCAAGTGG-3'
	Reverse primer	5'-GGAGGCATTGCTGATGATCT-3'

Supp Table 2*: Top ten of down- (negative logFC) and up regulated (positive logFC) genes after whole genome sequencing of K562 vs OCI-AML3 cells upon treatment with OTX015.*

Position	Gene Symbol	logFC	P.Value
TC01003403.hg.1	SLAMF6	-3.49147	5.89451118193039e-34
TC01001640.hg.1	PTPRC	-2.6289666667	1.8535949882457e-29
TC01003378.hg.1	SPTA1	-2.4937166667	9.28557583175231e-33
TC02000623.hg.1	IL18RAP	-2.33878	2.1204427293552e-25
TC01001987.hg.1	C1orf100	-2.1916	1.84952280601161e-20
TC15000796.hg.1	TM6SF1	-2.13317	6.83392932074306e-18
TC02000954.hg.1	GALNT5	-2.0946066667	1.07462730511599e-26
TC06000647.hg.1	TFAP2B	-2.06752	1.02143852061162e-20
TC14000902.hg.1	RNASE1	-2.0380533333	9.28053268825489e-23
TC12001718.hg.1	PTPRB	-2.0196733333	6.4168889993847e-30
TC15000945.hg.1	ARRDC4	4.0546766667	6.08821172467652e-26
TC17000796.hg.1	SNORA38B	3.03333	2.64055457057049e-20
TC07001562.hg.1	HGF	2.27311	5.24878344845297e-23
TC02002499.hg.1	SCN9A	2.1551933333	4.75263267193313e-26
TC18000554.hg.1	BCL2	1.9623066667	1.72660433612918e-16
TC11001368.hg.1	OR2D2	1.8800366667	4.49844104394644e-12
TC03001908.hg.1	IGSF10	1.8667866667	1.11715012616426e-18
TC07000780.hg.1	RNA5SP243	1.86289	1.28876588216316e-09
TC02001614.hg.1	WDR35	1.7575266667	1.78750821933943e-17
TC07001294.hg.1	TARP	1.7279666667	2.54635607648742e-15

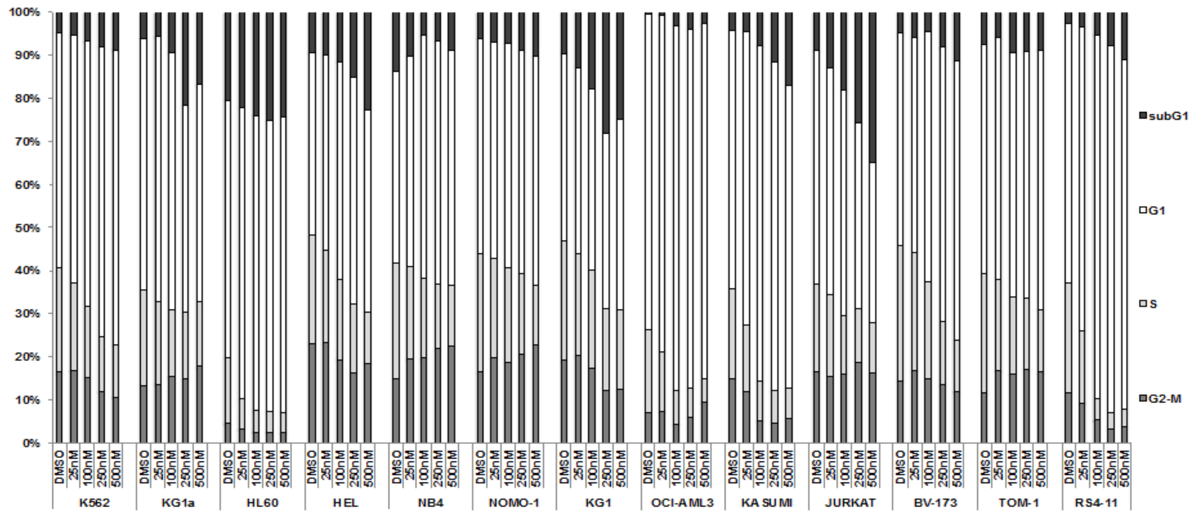

Supp Figure 1: Effects of OTX015 on the cell cycle. Cell cycle alterations at 48 h induced after OTX015 25 nM-500 nM in leukemia cell lines: X-axis, cell lines, Y-axis, cells in terms of percent cells in sub-G1, G1, S and G2/M phases. Results are shown as mean from duplicates of three independent experiments.

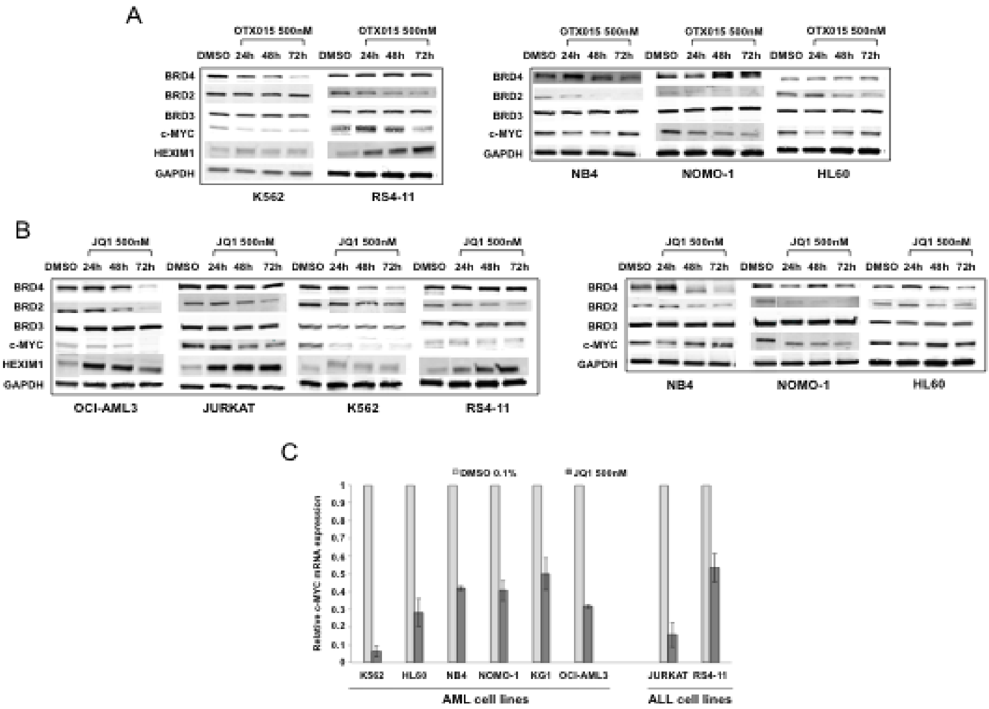

Supp Figure 2: Effects of OTX015 and JQ1 on c-MYC, BRD2/3/4 and HEXIM1 protein expression and of JQ1 on *c-MYC* mRNA in AML and ALL cell lines. Western blots showing BRD2/3/4, c-MYC and HEXIM1 expression in additional cell lines after treatment for 24, 48 or 72h with **(A)** 500nM OTX015, **(B)** 500nM JQ1**,** or 0.1% DMSO. GAPDH was used as a loading control. One representative experiment out of three is shown. **(C)** RT-qPCR showing *c-MYC* downregulation in AML and ALL cell lines after 48h exposure with 500nM JQ1, relative to *GAPDH* normalized to 0.1% DMSO.

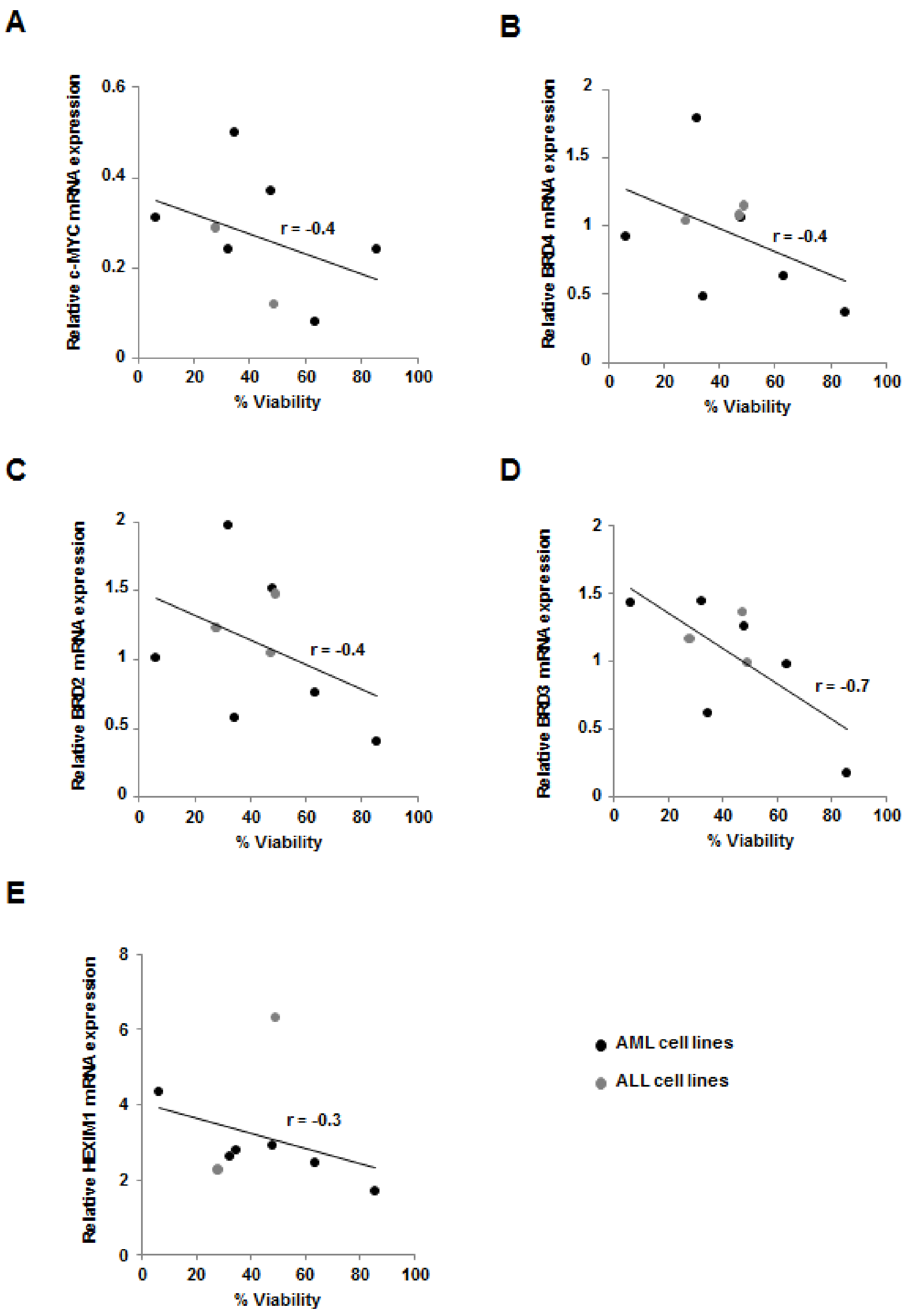

Supp Figure 3: Correlation between cell viability and mRNA expression of selected genes. Pearson's correlation test between the percentage of viability in cells treated by 500nM OTX015 and *c-MYC* (A), *BRD4*, *BRD2*, and *BRD3* (B-D), *HEXIM1* (E) mRNA expression.

A

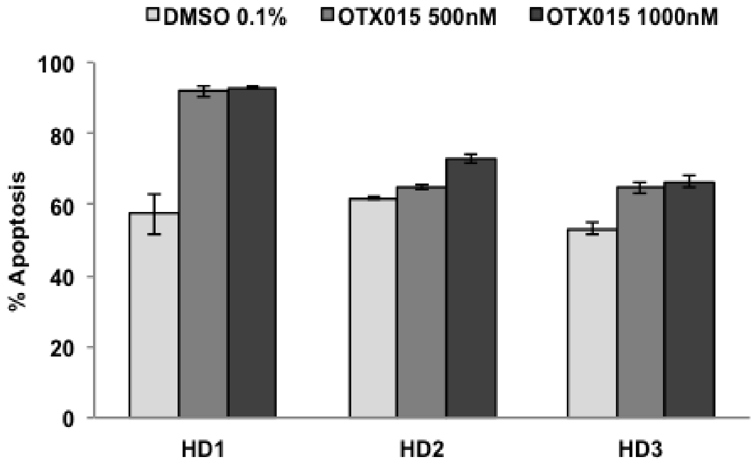

B

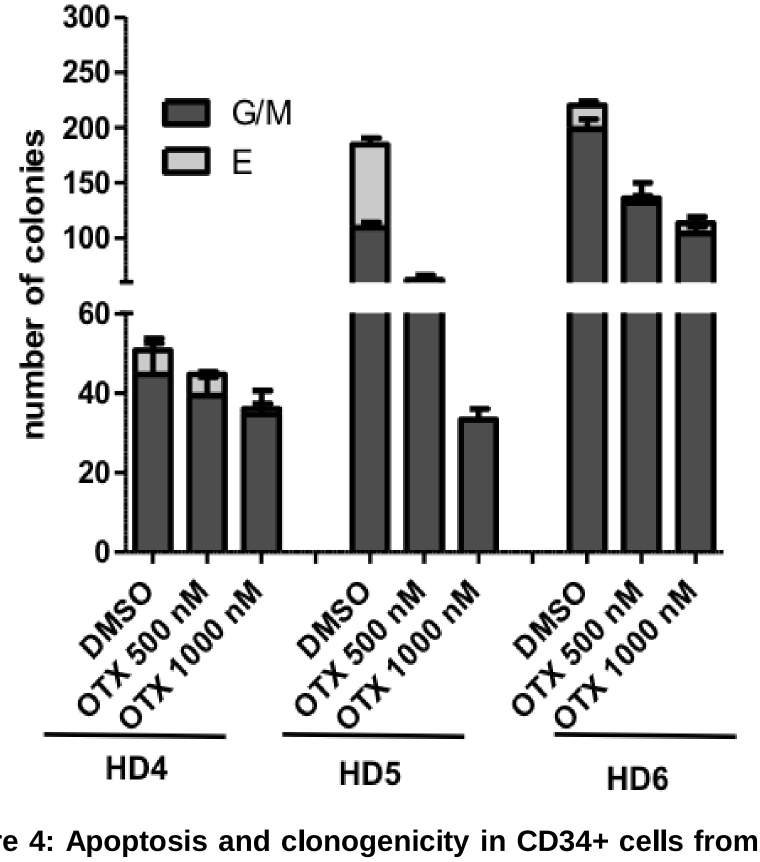

Supp Figure 4: Apoptosis and clonogenicity in CD34+ cells from healthy donors after exposure to OTX015. (A) CD34+ cells were exposed for 72h to OTX015 and apoptosis was determined by AnnexinV/PI. **(B)** Clonogenic assays performed in cytokine-supplemented methylcellulose after treatment with OTX015 or DMSO. G/M=Granulocyte/Macrophage; E=Erythroïd; HD=healthy donor

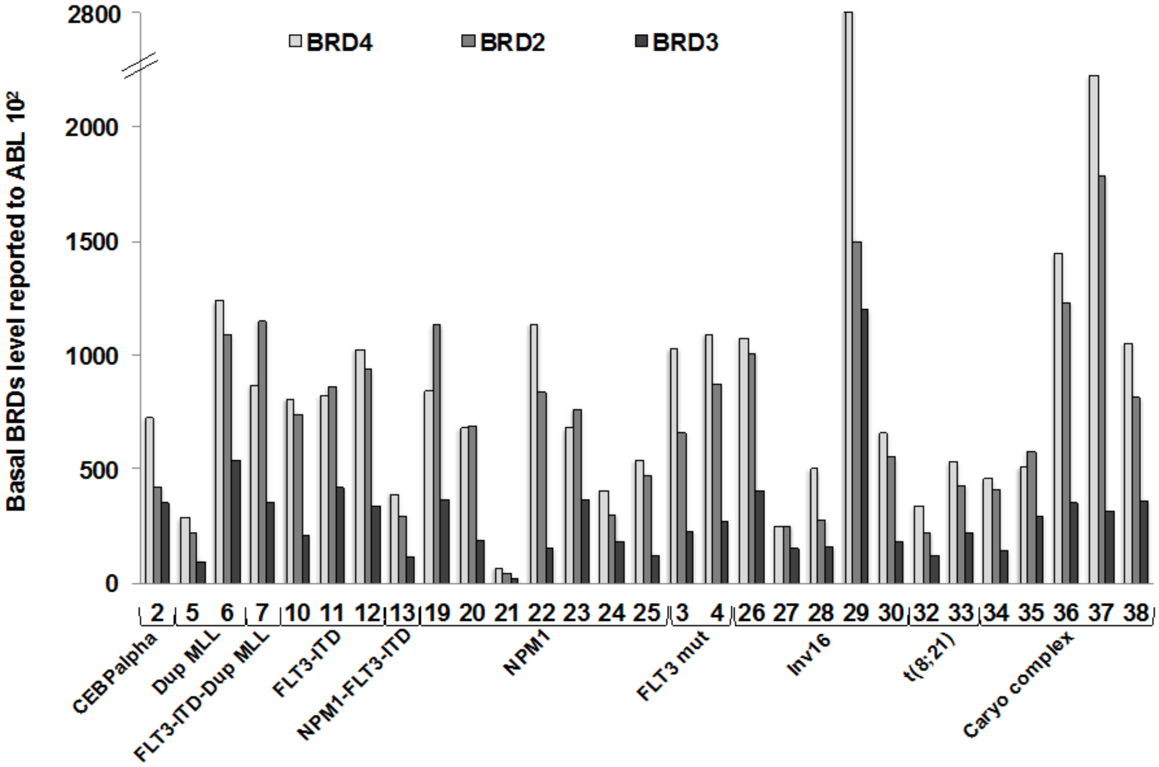

Supp Figure 5: Basal gene expression of *BRD2/3/4* in AML patient samples. Expression is shown for 29 AML patient samples**.** X-axis, patient samples. Y-axis, mRNA quantities, relative to *ABL*10^2.

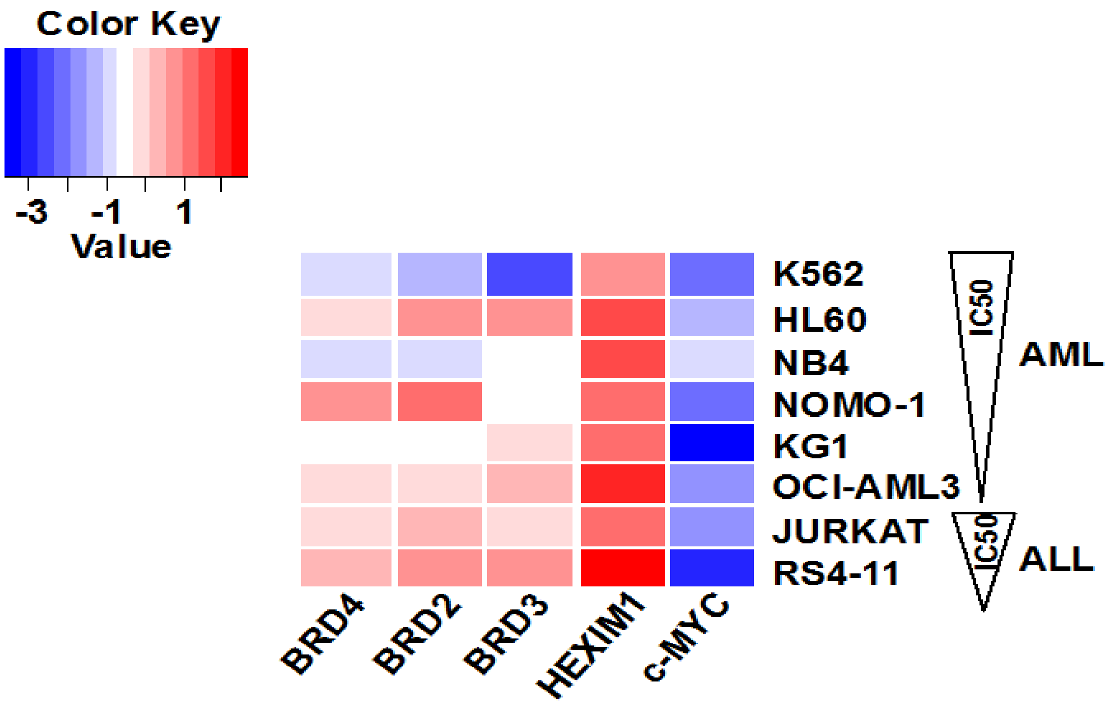

Supp Figure 6: Heatmap of gene expression after treatment with OTX015 at 500 nM. Modulation of *c-MYC*, *BRD4*, *BRD2*, *BRD3* and *HEXIM1* after OTX015 exposure at 500 nM. Repression in blue. Overexpression in red.

Supplementary Methods

Peripheral blood nucleated cells from apharesis products of healthy donors obtained after informed consent were enriched for CD34+ cells by immunomagnetic selection (MACS®, Miltenyi, Germany). Cells were grown in suspension in RPMI medium supplemented with 10% FCS, and 2 million cells were plated in 6-well plates, and treated at day 1 with either DMSO 0.1% or 500nM or 1000nM OTX015. At day 4, cells were numbered, and equal numbers of cells (500 cells/mL of methylcellulose) were seeded in triplicate in methylcellulose (METHOCULT® H4100, Stem Cell Technologies, Grenoble), supplemented with 30% FCS (Stem Cell Technologies, Grenoble) and cytokines: SCF 25 ng/mL, IL-3 10 ng/mL, G-CSF 10 ng/mL, GM-CSF 10 ng/mL and EPO 3 IU/mL (all from Peprotech France, Neuilly sur Seine).
